# Predicting Grade and Patient Survival in Renal Cancer Using Machine Learning Analysis of Nucleolar Prominence

**DOI:** 10.1002/cam4.71196

**Published:** 2025-09-09

**Authors:** Elena Ivanova, Alexey Fayzullin, Victor Grinin, Dmitry Zhavoronkov, Dmitry Ermilov, Maxim Balyasin, Anna Timakova, Alesia Bakulina, Yusif Osmanov, Ekaterina Rudenko, Alexander Arutyunyan, Ruslan Parchiev, Nina Shved, Marina Astaeva, Aleksey Lychagin, Tatiana Demura, Peter Timashev

**Affiliations:** ^1^ Institute for Regenerative Medicine Sechenov First Moscow State Medical University (Sechenov University) Moscow Russia; ^2^ B.V.Petrovsky Russian Research Center of Surgery Moscow Russia; ^3^ World‐Class Research Center “Digital Biodesign and Personalized Healthcare, Sechenov First Moscow State Medical University (Sechenov University) Moscow Russia; ^4^ PJSC VimpelCom Moscow Russia; ^5^ Scientific and Educational Resource Center Peoples' Friendship University of Russia Moscow Russia; ^6^ Institute of Clinical Morphology and Digital Pathology, Sechenov First Moscow State Medical University (Sechenov University) Moscow Russia; ^7^ Medical Neuronets Moscow Russia; ^8^ UNIM, Skolkovo Innovation Center Moscow Russia; ^9^ Department of Faculty Surgery Sechenov First Moscow State Medical University (Sechenov University) Moscow Russia; ^10^ Department of Trauma, Orthopedics and Disaster Surgery Sechenov First Moscow State Medical University (Sechenov University) Moscow Russia

**Keywords:** artificial intelligence, computational pathology, computer vision, digital pathology, renal cell carcinoma

## Abstract

**Background:**

Patients with clear cell renal cell carcinoma (ccRCC) often undergo organ resection, with treatment strategies based on recurrence risk. Current metastatic potential assessments rely on the WHO/ISUP grading system, which is subject to interobserver variability.

**Methods:**

We developed an artificial intelligence (AI) model to classify cells according to contemporary grading rules and evaluated the prognostic significance of tumor cell profiles, particularly focusing on cells with prominent nucleoli.

**Results:**

The model accurately distinguished low (G1/G2) and high (G3/G4) grades, achieving an area under the ROC curve of 0.79. Survival analysis identified four tissue patterns defined by total cell density and the proportion of cells with prominent nucleoli. The relative abundance of such cells had greater prognostic value than their mere presence, correlating with survival times ranging from 2.2 to over 6 years. Additionally, we confirmed that dystrophic changes and focal necrosis are linked to shorter survival.

**Conclusion:**

These findings suggest that incorporating refined criteria into the WHO/ISUP system could enhance its prognostic accuracy in future revisions.

## 1 Introduction

Renal cell carcinoma (RCC), originating from renal tubular epithelial cells, has the highest mortality rate among genitourinary cancers, with incidence increasing by2% to 4% annually [1, 2]. In 2024, an estimated 81,610 new RCC cases and 14,390 related deaths will occur in the United States [1]. RCC encompasses various histological types with distinct genetic, biological, and behavioral traits. Clear cell renal cell carcinoma (ccRCC) is the most common subtype, accounting for65% to 80% of cases [1, 2, 3].

Pathological examination remains critical for diagnosing primary lesions and metastases, guiding treatment, and assessing recurrence risk. The World Health Organization/International Society of Urological Pathology (WHO/ISUP) system grades tumors from 1 to 4 based on nucleolar prominence and other features [4, 5]. Post‐operative management varies by grade: patients with Grades 1 to 2 often undergo partial nephrectomy with periodic imaging, while Grades 3 to 4 cases may require radical nephrectomy and additional treatments such as targeted therapy or immunotherapy. Follow‐up intervals are more frequent in higher‐grade cases due to increased metastasis risk. However, challenges such as interobserver variability and labor‐intensive manual assessments limit the system's effectiveness.

Artificial intelligence (AI) in medical imaging offers advantages over traditional methods [6, 7]. Computer vision can automate tumor grading and identify diagnostic patterns such as necrosis or vascular invasion [8, 9, 10]. Automated grading systems for RCC, including adaptations of WHO/ISUP and Fuhrman criteria, have effectively distinguished between low and high‐grade cases [11, 12, 13]. Yet, the predictive value of these systems is limited by the discrete nature of existing grading schemes, designed for practical application by pathologists.

Our study seeks to enhance ccRCC grading through AI‐based analysis of tumor tissue cellular composition. We evaluated whether our metrics could serve as histological biomarkers to predict expert‐assigned grades within the WHO/ISUP framework. Additionally, we analyzed the relationship between these metrics and patient survival outcomes. By leveraging computer vision to detect subtle morphological features, we aim to propose refined grading criteria, contributing to future revisions of the WHO/ISUP system.

## 2 Materials and Methods

### 2.1 Dataset Preparation

All methods were carried out in accordance with relevant guidelines and regulations. All experimental protocols were approved by Sechenov University (Moscow, Russia). Informed consents were obtained from all subjects and/or their legal guardian(s) prior to having their data used in this study.

For this study, we included histological images and clinical data of patients from three independent sources: The Cancer Genome Atlas (TCGA), pathomorphological digital laboratory UNIM (Moscow, Russia) and the Institute of Clinical Morphology and Digital Pathology of Sechenov University (Moscow, Russia). We used only one histological slide image from each case. The internal datasets included 49 tumor samples from UNIM and 83 tumor samples from the Institute. All formalin‐fixed paraffin‐embedded tumor samples were sectioned, stained with H&E, and digitized with a Leica Aperio AT2 scanner (Leica Microsystems, Wetzlar, Germany) at ×40 magnification to obtain whole slide images (WSIs). The samples were anonymized and did not include any clinical information.

Additionally, we used 105 cases of ccRCC from TCGA as the external dataset, using 70 of these cases to evaluate the prognostic value of the model calculations. In total, we selected 167 ccRCC WSIs with Fuhrman grades ranging from 1 to 4 for analysis. The images were blindly reviewed in the .svs file format using Case Viewer software (3DHistech, Hungary) and reclassified according to the WHO/ISUP classification.

**FIGURE 1:**
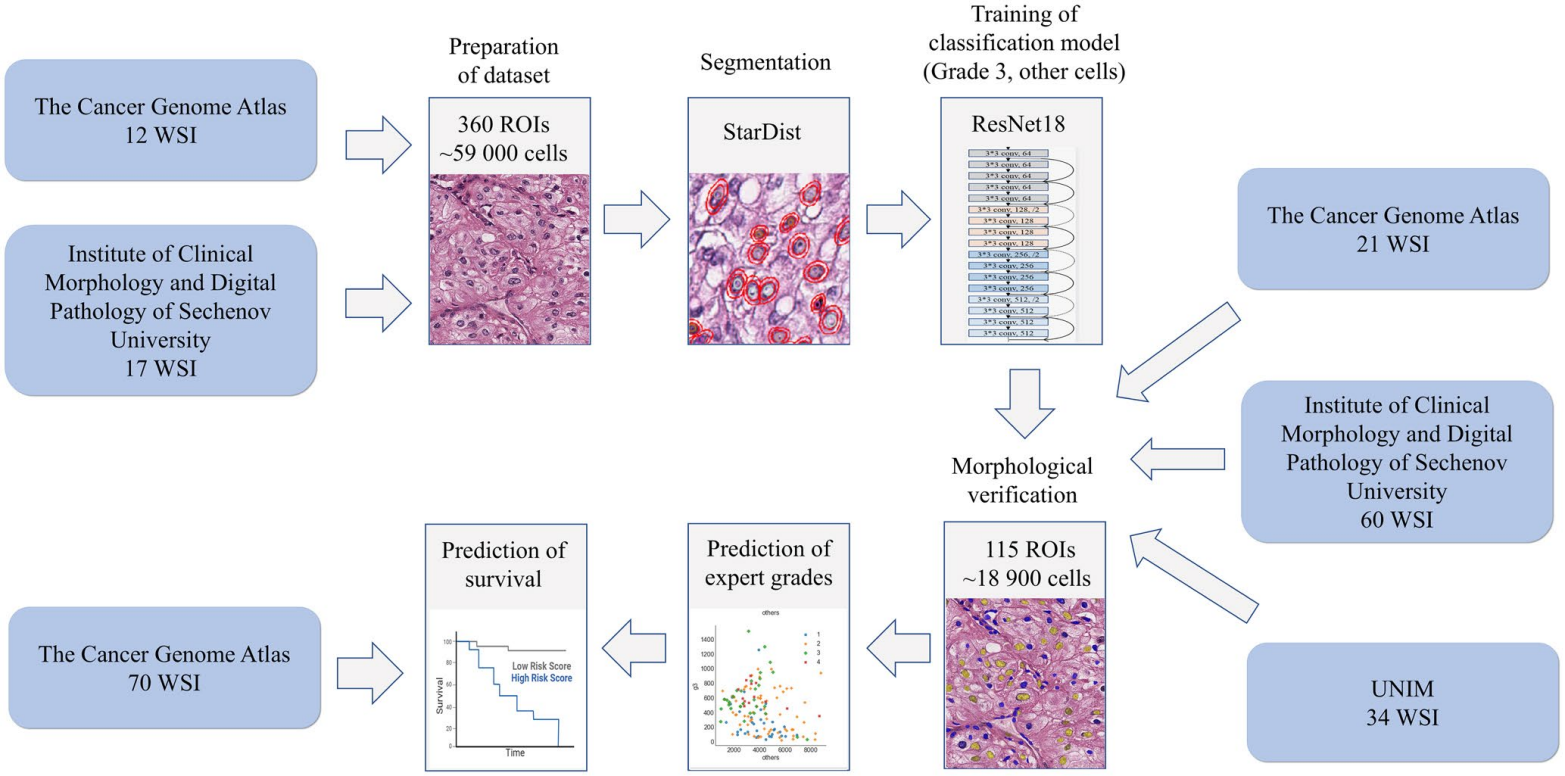
Study design. For training, we obtained histological images of patients with clear cell renal cell carcinoma (ccRCC) from The Cancer Genome Atlas (TCGA) and the Institute of Clinical Morphology and Digital Pathology of Sechenov University. We used the Stardist model for segmentation of nuclei, which were annotated by experts and used for training of the ResNet18 classification model. We conducted verification using 115 slide images from three datasets (TCGA, UNIM and Sechenov University). The model's performance was evaluated using ROC analysis. Survival prediction was assessed using 70 WSIs from TCGA, divided into two groups based on patient survival, and analyzed using the Kaplan–Meier method and Log‐rank test.

After applying inclusion and exclusion criteria, our final dataset comprised 144 WSIs with various grades of ccRCC. The inclusion criteria were: (1) patient age over 18 years; and (2) a confirmed diagnosis of ccRCC. The exclusion criteria were: (1) the presence of other diagnoses coded with “C” in the International Classification of Diseases, 10th Revision (ICD‐10); (2) the presence of artifacts in the images; and (3) poor quality of sample preparation. The distribution of images by grade is shown in Table 1. We depicted the design of our study in Figure 1.

**TABLE 1:** Characteristics of the dataset.

	TCGA (external dataset)	UNIM (internal dataset)	Institute of Clinical Morphology and Digital Pathology (internal dataset)
*Training set*			
Grade 1	1	—	5
Grade 2	7	—	7
Grade 3	1	—	4
Grade 4	3	—	1
Total WSIs (*n*)	12	—	17
*Prediction of grade (validation set)*			
Grade 1	1	6	20
Grade 2	12	13	28
Grade 3	2	14	10
Grade 4	6	1	2
Total WSIs (*n*)	21	34	60
*Prediction of survival (verification set)*			
Grade 1	—	—	—
Grade 2	46	—	—
Grade 3	7	—	—
Grade 4	17	—	—
Total WSIs (*n*)	70	—	—

### 2.2 Segmentation Model

The Stardist model was used for a segmentation–classification pipeline for its superior ability to identify nuclei boundaries in H&E slide images [14, 15]. This model predicts the distance (*r*) to the boundary of an object from a fixed set of rays and the probability (d) of each object, creating a comprehensive set of possible polygon candidates for a given image. Non‐maximum suppression is applied to these candidates to achieve the final segmentation result. The Stardist extension was installed in QuPath v.0.4.3 to assist during annotation. The segmented nuclei and expert annotations were then used to train the classification model [14, 15].

### 2.3 Annotation and Training of Classification Model

To train the classification model, we used 12 WSIs from the TCGA and 17 WSIs from the Institute of Clinical Morphology and Digital Pathology. Regions of interest (ROIs) were selected using the QuPath v.0.4.3 program and exported as .json files. These ROIs included tumor tissue containing both tumor and stromal cells while excluding areas of diffuse necrosis and regions with poor focus during scanning. We selected10 to 15 squares with a side length of 500 μm for further annotation in each WSI. We randomly divided the ROIs into training, validation, and test sets, resulting in 277, 51, and 32 unique ROIs, respectively. Using the Stardist extension, we identified polygon borders of cells, extracted each cell image, and used them along with associated labels to train the ResNet18 classification model.

For training and validation, we classified each cell in 328 ROIs as one of five classes: G1, G2, G3, G4, and other cells. Experts assigned cells with small, clearly defined nuclei without visible nucleoli to G1. Cells with an eosinophilic, rounded nucleolus noticeable at magnifications from ×400 to ×100 in the center of the nucleus were classified as G2. G3 cells had large basophilic nuclei with clear or slightly irregular round‐shaped dark purple or light eosinophilic nucleoli that were clearly visible at ×100 magnification. Giant multinucleated tumor cells and cells with rhabdoid or sarcomatoid features were labeled as G4. Two experienced pathologists specializing in onco‐ and uropathology annotated all cells inside the ROIs using QuPath v.0.4.3 software. A third experienced pathologist then verified the annotations on each slide to ensure accuracy. As a result, we comprised a dataset based on the WHO/ISUP classification. “Other cells” class included both stromal cells and tumor cells that could not be clearly assigned to a specific grade class. We included G4 cells in the “Other cells” class due to their low content, less than a dozen in an average Grade 4 ROI. Classification errors occurred mostly between tumor cells and other cells.

For the test set, we applied a simplified four‐class annotation (G1, G2, G3 and other cells) across 32 ROIs to evaluate how the trained model performed in predicting cell classes. The key task in this evaluation was to classify between Grade 3 cells and Others, which included all stromal and tumor cells lacking prominent nucleoli. Table 2 shows the distribution of cells of different classes in the training, validation, and test sets of cell images.

**TABLE 2:** Class distribution in training, validation and test sets.

Class	Training set	Validation set	Test set
	Number of cells, %	Number of cells, %	Number of cells, %
G1	5420, 11.82	816, 12.55	1101, 14.72
G2	6295, 13.73	764, 11.75	4452, 3.97
G3	1075, 2.34	127, 1.95	271, 65.19
Other cells	33,065, 72.11	4793, 73.75	1005, 16.12

We used 41,341 cells for training, 5411 cells for validation, and 5824 cells for testing, including 271 Grade 3 cells. The training process involved using a pre‐trained ResNet18 model to classify cells into two categories: Grade 3 and Others. Training lasted for 100 epochs, with early stopping if validation quality did not improve for 10 consecutive epochs. The model from the epoch with the highest validation quality was selected.

We had two data sets of tumor cells for the calculation of accuracy, specificity, and recall of the trained models. The validation metrics for the classification were the *F*1 score and the absolute difference between validation precision and recall. We used the Adam optimizer with parameters: learning rate = 0.002, weight decay = 1e−4, and batch size = 128. Image augmentation, such as color changes and blurring, was used to improve results, but not during testing.

To ensure the consistency and accuracy of the data and ground‐truth labels, several quality control measures have been incorporated into the AI training process [16, 17]. This includes standardized preprocessing steps, such as color normalization, image blurring, and brightness variations, which are aimed at enhancing the generalizability of the model. It is important to note that the augmentations were only applied during training, and not during testing, to maintain the integrity of the evaluation process. Prior to ROI selection, all WSIs underwent a quality assessment. Slides with poor staining, imaging artifacts, or other defects were excluded based on predetermined criteria to ensure only high‐quality data was used in training. To ensure the reliability of the annotations, a multi‐stage expert validation process was implemented. Two experienced pathologists with expertise in oncological and urological pathology independently annotated the ROIs using QuPath version 0.4.3 software. A third senior pathologist then reviewed and verified all annotations in order to minimize errors and indirectly evaluate interobserver agreement.

In summary, our trained model used ROI as input, segmented cell nuclei, used masks to extract cells from the original image, and classified each cell. The output included a list of coordinates, classes, and probabilities for each cell, which could be imported into image analysis software like QuPath.

### 2.4 Verification

For verification, we used 115 WSIs from three datasets: TCGA (*n* = 21), UNIM (*n* = 34) and the Institute of Clinical Morphology and Digital Pathology (*n* = 60). In each slide, we selected one ROI with a perimeter of 4000 μm with400 to 1200 cells. Pathologists reviewed these ROIs with segmentation masks generated by the deep learning model, classifying tumor cells into two classes: Grade 3 and others.

The specificity and sensitivity of the deep learning model's predictions were evaluated using the receiver operating characteristic (ROC) curve and the area under the ROC curve (AUC).

The statistical analysis of the experimental quantitative data was performed using GraphPad Prism version 10.2.3.403 for Windows (GraphPad Software Inc., San Diego, CA, USA) and R v4.1.2, with the *betareg* v3.1–4, *car* v3.0‐13, and *emmeans* v1.7.3 libraries. ROC analysis was performed to compare the features, and Youden's J index was calculated. The number of G3 cells in 1 mm^2^ and the percentage of G3 cells were analyzed by the one‐way ANOVA method followed by Tukey's multiple comparison test. The statistical analysis results were presented as boxplot graphs. Two‐dimensional visualization of the calculated values was done using Python v.3.10 and NumPy v.1.21.1 and pandas v.1.4.1 libraries. Statistical significance was set at *p* < 0.05.

### 2.5 Survival Prediction

To assess survival, we used 70 WSIs with accompanying clinical information from the TCGA database. This dataset included 20 women and 50 men; the average age was 61.14 ± 12.54; the stages of the disease were distributed as follows: 1st was observed in 27 patients (38.57%), 2nd—in 6 patients (8.57%), 3rd—in 17 patients (24.28%), 4th—in 18 patients (28.58%). These samples were divided into two groups: 35 from patients who died within 5 years of diagnosis and 35 from patients who survived beyond this period. The dataset included patients with ccRCC of Grades 2, 3, and 4. The slides were reviewed by an experienced pathologist and reclassified according to the WHO/ISUP system. We processed the WSIs using the trained model to segment and classify cells into two classes (Grade 3 and others).

From each slide, we selected ROIs with a perimeter of 4000 μm containing 400 to 1200 cells. To evaluate the relationship between cellular composition in the tumor and patient survival, we divided the data into subgroups using two thresholds for the G3 variable and other variables, determined by Youden's J index. This allowed us to compare the survival of patients with different tumor compositions. The cut‐offs were 533 and 4133 for cells with prominent nucleoli (G3) and Others, respectively. Based on these thresholds, we formed four subgroups corresponding to four quadrants: (1) G3^+^/Others^−^; (2) G3^+^/Others^+^; (3) G3^−^/Others^−^; (4) G3^−^/Others^+^.

In each quadrant, survival was evaluated using the Kaplan–Meier method with the Log‐rank test (Mantel‐Cox test). The distribution of patient samples by the obtained quadrants according to the grade was evaluated using Fisher's exact test.

To ensure methodological rigor and avoid potential circularity in downstream analyses, the dataset was divided into three independent subsets: (1) a training cohort used to develop the deep learning model, (2) a validation cohort used to assess model performance and optimize classification thresholds, and (3) a survival analysis cohort used exclusively for evaluating the prognostic relevance of model‐derived features. No overlap occurred between these subsets.

The threshold for the proportion of cells with prominent nucleoli—used to define distinct tissue patterns in the survival analysis—was determined using Youden's J index on the validation cohort. This approach maximized the trade‐off between sensitivity and specificity for distinguishing low‐grade and high‐grade cases. The resulting cutoff was then applied only to the survival cohort, ensuring full independence from the data used during model training and threshold selection. This strategy was employed to minimize the risk of circularity and overfitting in survival analysis.

## 3 Results

### 3.1 Classification Model Performance

We verified the model's performance on WSIs of different grades of renal cancer and compared its accuracy with expert assessments on two validation and test sets. The statistical metrics for the validation set are as follows: accuracy: 0.88; precision: 0.60; recall: 0.51; *F*1‐score: 0.56 (Figure S1). For four‐class test set, we achieved the following metrics: accuracy: 0.96; precision: 0.87; recall: 0.87; *F*1‐score: 0.87. The algorithm misclassified some tumor cells as Grade 3, mostly cells that were annotated as Grade 2. However, when these cells were analyzed by pathologists later, they described them as cells with large stellate‐shaped nucleoli or blurred borders (Figure S2). These improved metrics indicated that the model was capable of robust identification of cells with prominent nucleoli, prompting us to investigate the prognostic value of these metrics (Figure S3).

### 3.2 Verification of the Classification Model

**FIGURE 2:**
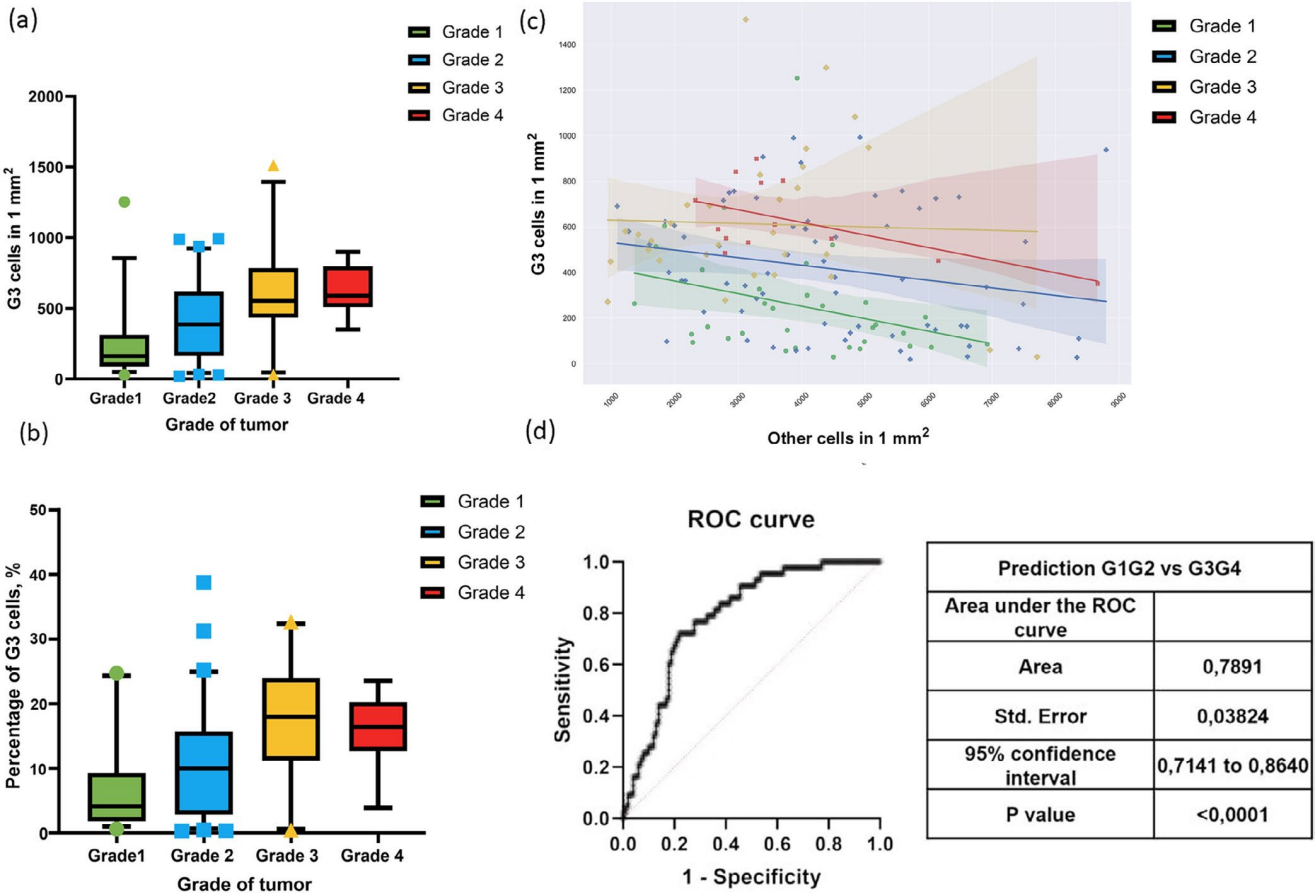
Statistical analysis of cell profile and expert grade predictions. (a) Density of Grade 3 cells per 1 mm^2^ in tumor tissue. The boxplot (5–95 percentile) graph illustrates the spatial distribution of Grade 3 cells within the tumor tissue, highlighting areas with a high density of these cells. (b) Percentage ratio of Grade 3 cells in tumor tissue. The boxplot (5–95 percentile) graph illustrates the percentage ratio of Grade 3 cells relative to the total cell number in the tumor tissue, providing a quantitative measure of their prevalence. (c) Correlation between Grade 3 cell ratio and tumor grade. This figure depicts the relationship between the total number of Grade 3 and other cells to the tumor grade determined by expert pathologists. The highest number of Grade 3 cells was seen in Grade 3 tumors, while Grade 4 tumors showed a reduction in Grade 3 cells due to the emergence of cells with atypical morphology. (d) ROC analysis for predicting Grade1 to 22 vs. Grade3 to 44. The ROC curve illustrated the trade‐off between sensitivity and specificity in distinguishing between lower‐grade (Grade 1–2) and higher‐grade (Grade 3–4) RCC cases. The curve demonstrated the model's ability to differentiate between these grades, with the area under the curve indicating the overall performance of the classification.

We investigated the correlation between the ratio of Grade 3 cells to the total number of cells and the overall tumor grade as determined by expert pathologists. The analysis of total number and percentage of G3 cells revealed statistically significant differences (total number of G3 cells: Grade 1 vs. Grades 2, 3 and 4, Grade 2 vs. Grade 3; percentage of G3 cells: Grade 1 vs. Grades 3 and 4, Grade 2 vs. Grade 3) or trends (total number of G3 cells: Grade 2 vs. Grade 4, *p* = 0.06; percentage of G3 cells: Grade 1 vs. Grade 2, *p* = 0.18, Grade 2 vs. Grade 4, *p* = 0.12) between all groups except Grade 3 and Grade 4 (Figure 2). Specifically, we observed the increase in G3 cells content from Grade 1 to Grade 3. However, in Grade 4 tumors, the proportion of Grade 3 cells did not increase. This trend suggests that as tumors progress to Grade 4, there is a shift in cellular morphology. Grade 4 tumors often exhibit characteristics such asbizarre nucleus, nuclear deformations, sarcomatoid or rhabdoid differentiation, and giant cell formation. These morphological changes complicate the classification process, as these atypical cells do not fit neatly into the categories used for lower‐grade tumors. The presence of such cells in Grade 4 tumors can lead to a decreased proportion of cells that can be straightforwardly classified as Grade 3.

The model demonstrated strong performance in binary classification between low and high‐grade cases, achieving an AUC of 0.79 ± 0.04. The model's sensitivity was 0.89, and its specificity was 0.59.

### 3.3 Tumor Cell Detection in Tissue Samples

**FIGURE 3:**
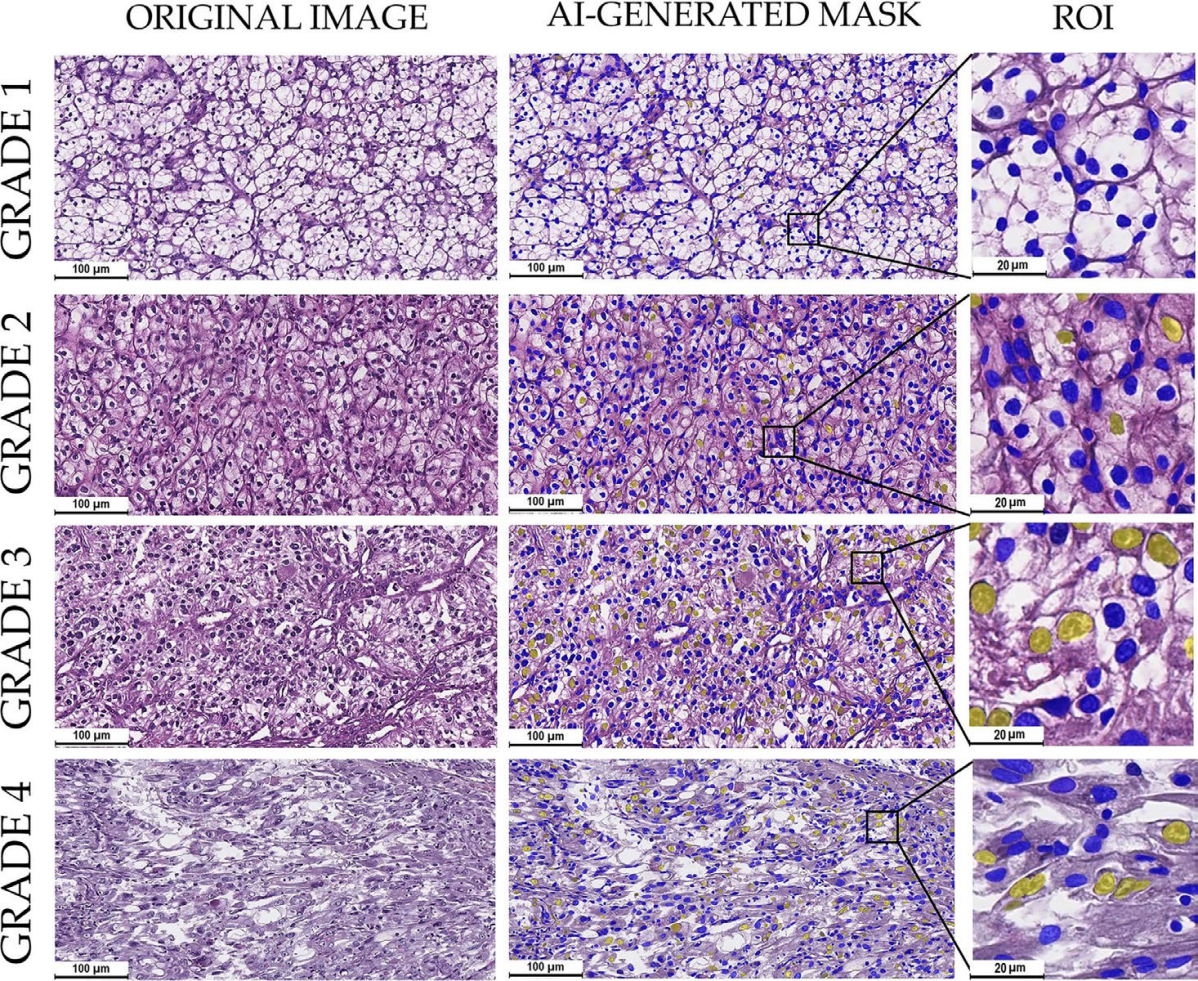
AI‐generated mask for slides of ccRCC with different grades (WHO/ISUP classification). Most tumor cells were highlighted with small oval blue masks in Grade 1 cases. However, singular cells were highlighted yellow visualizing rare tumor cells with prominent nucleoli. In Grade 2 tissue, a significant portion of tumor cells were highlighted with larger and polymorphic yellow masks. In some cases of Grade 3 samples, more than half of the tumor cells were highlighted with large yellow mask, these cells had very prominent nucleoli. These cells were also evident in Grade 4 slides, however, there were sparser than in Grade 3 cases.

Semi‐transparent masks overlaid original H&E images allowing for the assessment of Grade 3 tumor cell segmentation. Cells with prominent nucleoli were highlighted yellow, while stromal cells and cells with Grades 1, 2, and 4 were colored blue (Figure 3). The application of masks made it evident that Grade 3 cells are present in tumors of all WHO/ISUP grades; however, their numbers varied (Figure S3). Although in contradiction to the WHO/ISUP classification system, all renal cancers of Grades 1 and 2 included minimal or relatively small numbers of cells with prominent nucleoli.

**FIGURE 4:**
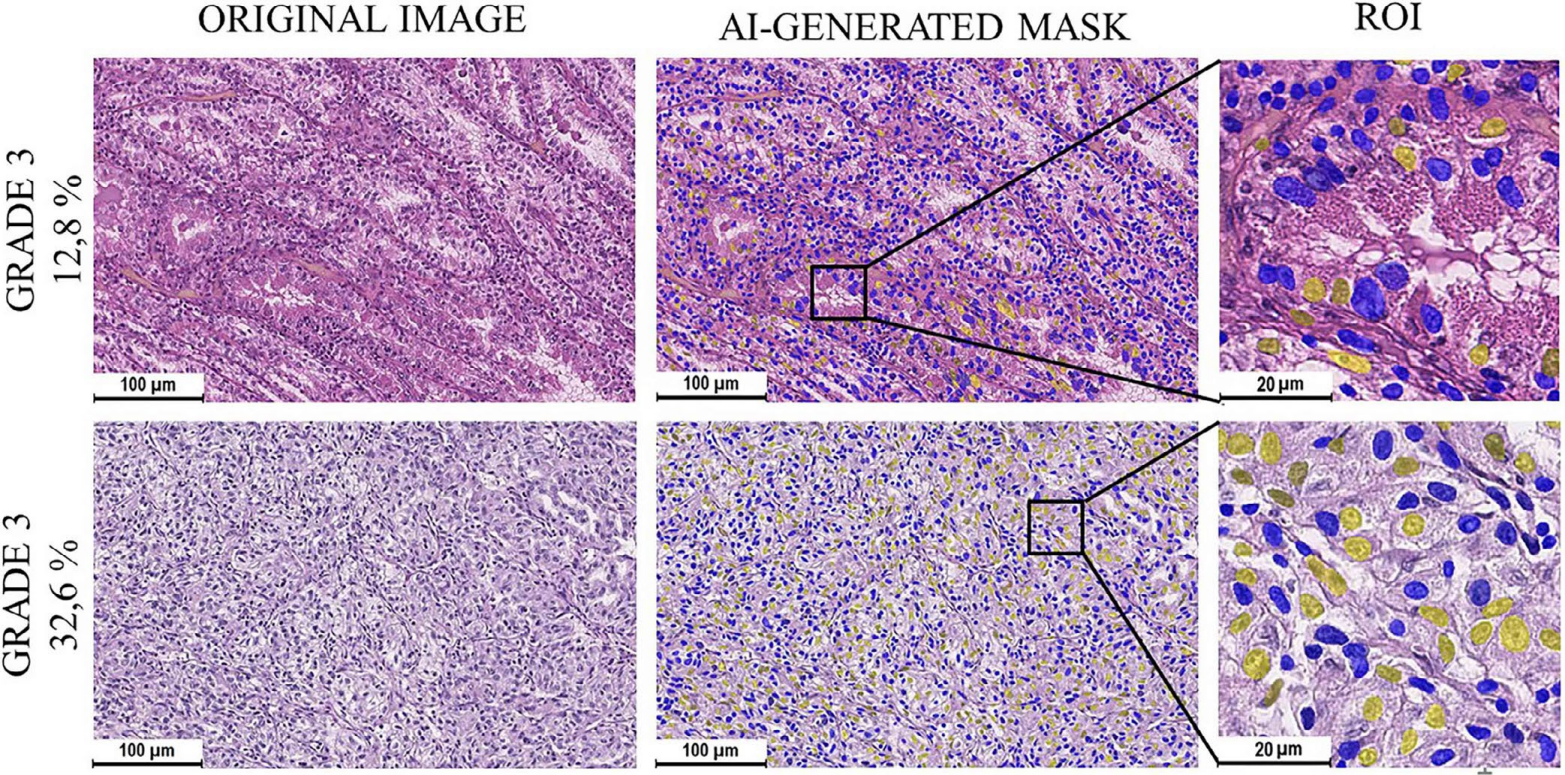
Variance of content of cells with prominent nucleoli (G3) in slides of Grade 3 ccRCC. The AI‐generated masks allowed to evaluate numbers and distribution of cells with prominent nucleoli. Two cases of ccRCC with similar nuclear grading presented approximately 3‐fold difference in relative number of G3 cells. G3 cells were highlighted yellow, while stromal cells and cells with Grades 1, 2 and 4 were colored blue.

The most interesting morphological finding was that there was a significant heterogeneity in the content of cells with prominent nucleoli in Grade 3 cases. The relative number of G3 cells varied from 0.39% to 32.62%. If we consider these cells indicators of metastatic potential of the tumor tissue, such differences in cellular profile make a case for identifying a diagnostic threshold (Figure 4).

### 3.4 Survival Analysis

We used metrics of G3 and Other cell distribution that we calculated by the model to investigate if they have an independent prognostic value. Based on the values of predicted G3 and Other cells per mm^2^ in tissue samples from a dataset containing patient survival data, two thresholds for the tumor tissues were identified: 533 G3 and 4133 Other cells per mm^2^. Using these thresholds, the dataset was divided into four quadrants:

1. Grade 3+/Other−: > 533 G3 cells and ≤ 4133 Other cells/mm^2^
2. Grade 3+/Other+: > 533 G3 cells and > 4133 Other cells/mm^2^
3. Grade 3−/Other−: ≤ 533 G3 cells and < 4133 Other cells/mm^2^
4. Grade 3−/Other+: ≤ 533 G3 cells and ≥ 4133 Other cells/mm^2^

**FIGURE 5:**
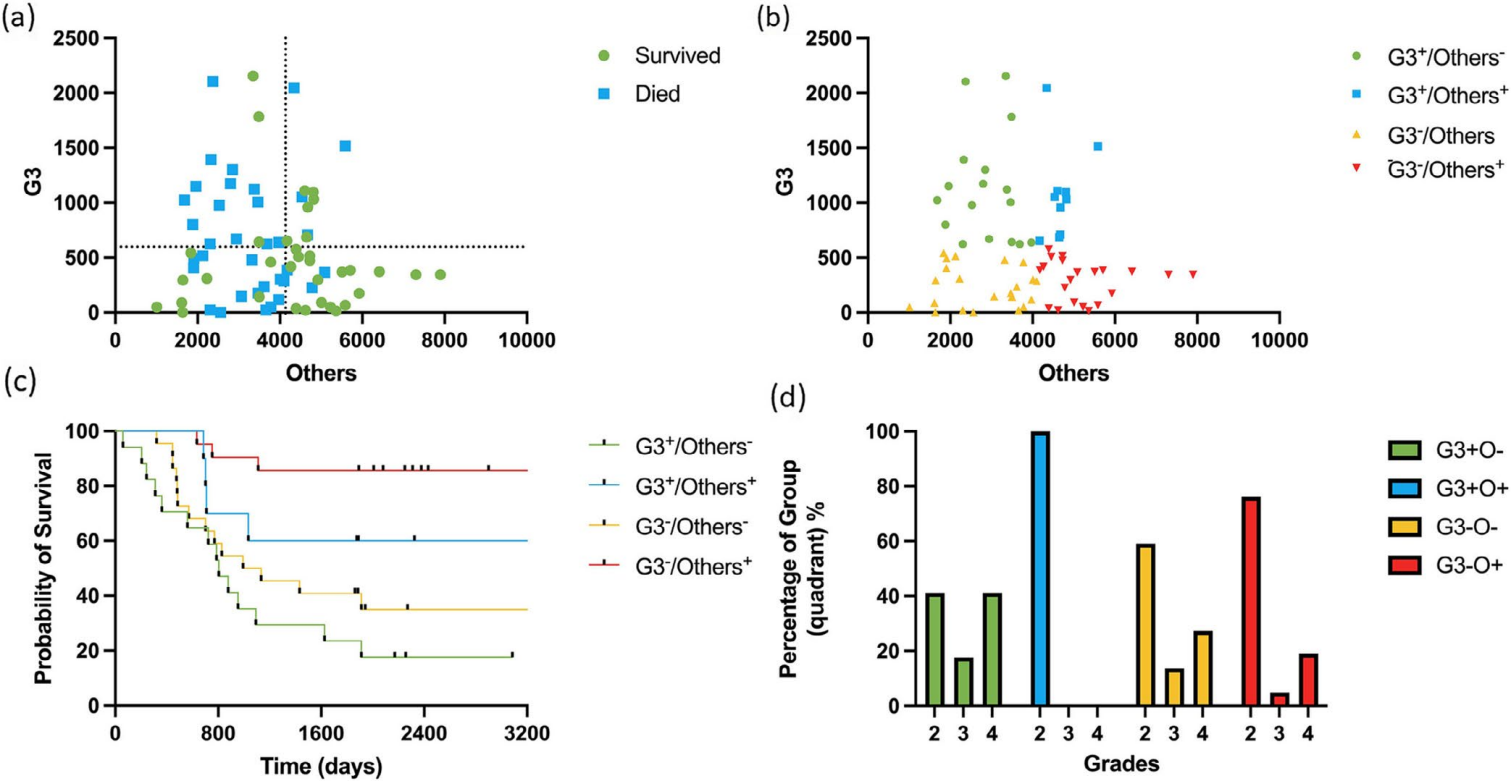
Statistical analysis of survival of patients with ccRCC. (a) Distribution of G3 and Other cell types among patients, depending on the outcome (color‐coded). Dotted lines represent the cut‐off values used to split the dataset into subgroups of survivors and deceased patients. (b) Distribution of quadrants based on the cut‐off values. (c) Kaplan–Meier survival analysis showing survival forecasts for each quadrant. (d) Distribution of quadrants by grade.

The median survival time for the entire dataset was 1912 days. Two groups, with a low number of Other cells, had a median time of life under 3 years. Patients in the Grade 3+/Other− quadrant had a median survival of 804 days, while those in the Grade 3−/Other− quadrant had a median survival of 1062.5 days. Two groups with a high number of Other cells were predicted to live more than 6 years. However, precise median survival times were not determined for them due to insufficient data (Figure 5).

Survival curves for the four quadrants demonstrated statistically significant differences (*p*‐value = 0.0005, log‐rank test), indicating distinct survival outcomes associated with quadrant membership. Additionally, the distribution of grades was significantly associated with belonging to a certain quadrant, as evidenced by a statistical trend (*p*‐value = 0.06349), which was confirmed by Fisher's exact test.

### 3.5 Morphological Description of Tumor Patterns

**FIGURE 6:**
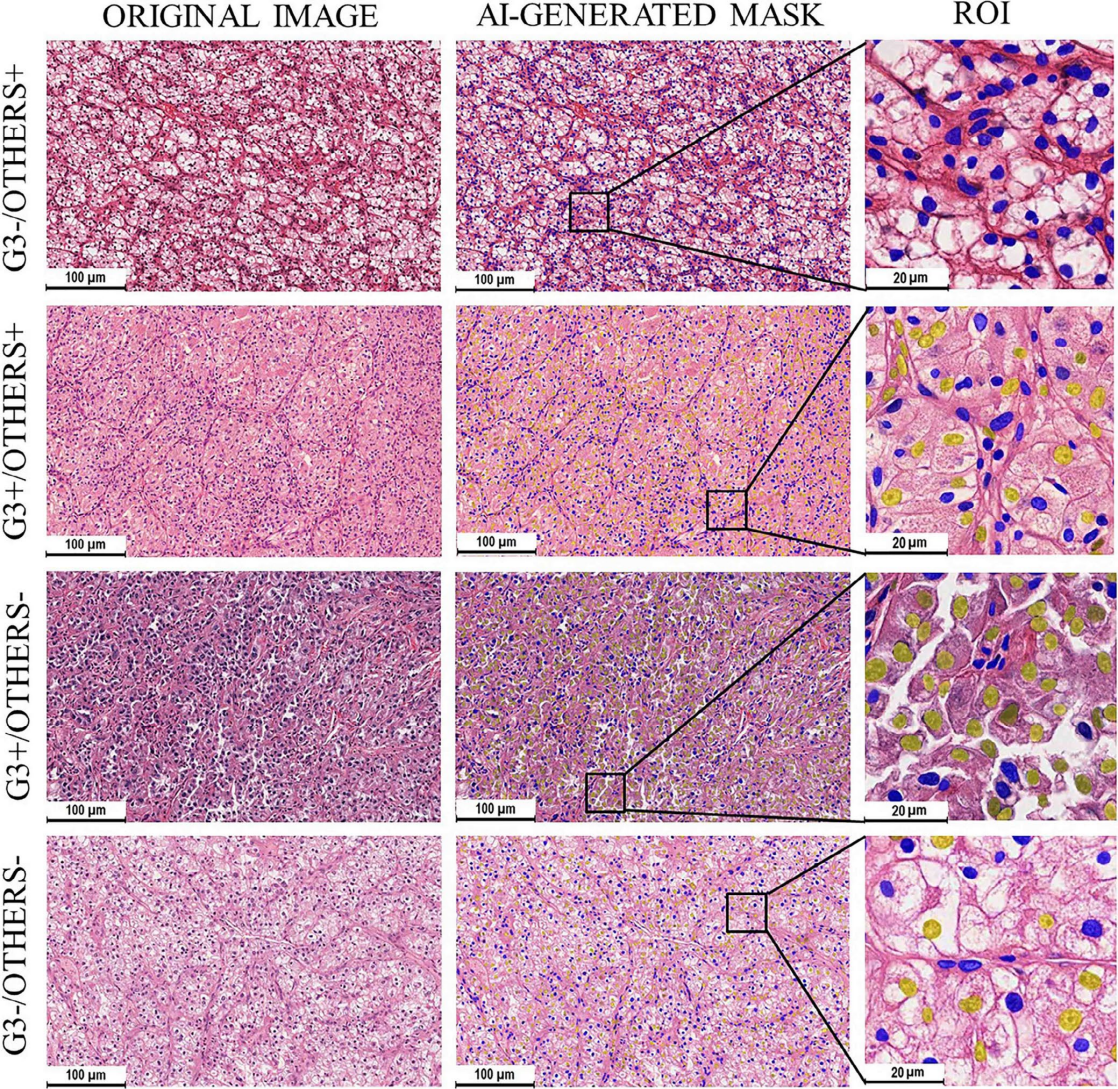
Histological analysis of ccRCC slides with 4 cellular patterns with highlighted cell with prominent nucleoli. The AI‐generated mask highlighted the distribution of G3 cells (yellow) and other cells (blue). This visualization aided in identifying distinct cellular patterns associated with different prognoses. In particular, upper two patterns were associated with better prognosis while lower two patterns had a prognosed survival under 3 years.

We identified a connection between tumor cellular composition and patient survival in ccRCC, prompting a reassessment of histological slides. Four distinct morphological patterns were defined based on the proportion of cells with prominent nucleoli and total cell density (Figure 6).

In the first (“monomorphic”) pattern, we identified a small number of G3 cells (5.24% ± 3.55%) while the number of other cells was high. This cell ratio was most often observed in Grade 2 tumors of patients who survived for a long time after diagnosis. In histological analysis, these tumors exhibited nested or alveolar growth patterns, tumor cells with optically clear cytoplasm and mostly uniform, small, basophilic nuclei with absent or inconspicuous nucleoli. This morphology aligned primarily with WHO/ISUP Grades 1 and 2, where nuclear atypia is minimal and nucleolar prominence is not a defining feature.

In the second (“pluralistic”) pattern, we observed increased numbers of both G3 cells and other cells. This cellular composition was present in both surviving and deceased patients, with survival times exceeding six years. Although expert pathologists classified these tumors as Grade 2, a notable proportion of G3 cells (18.45% ± 5.57%) was present. The growth patterns were alveolar, nested, or tubular, with rare cysts and hemorrhages; cells were round with clear cytoplasm and showed moderate variation in size and shape. Nuclei had slightly irregular borders, oval shape, light purple color, and nucleoli visible at ×400 magnification. The presence of G3 cells suggests a more aggressive tumor, yet experts classified them as Grade 2, possibly due to their low percentage. Despite the overall lower prevalence of nuclear atypia, strict following of the WHO/ISUP system requires grading all these cases as Grade 3.

In the third (“nucleolar”) pattern, we noticed the prevalent majority of G3 cells (28.29% ± 10.04%) and a small number of other cell types. Patients with this pattern had the shortest expected survival time, 2.2 years. Interestingly, these cases were more often evaluated as Grade 2 and Grade 4 than Grade 3. This highlights interobserver variability, a common challenge in ccRCC grading. The morphological examination confirmed tubular, nested, or alveolar growth patterns of tumor, presence of focal necrosis, hemorrhages, and singular cysts. The tumor cells had clear cytoplasm, and purple large nuclei with irregular borders with eosinophilic nucleoli were visible at low magnifications (×100). Some cells displayed signs of anaplasia and nuclear pleomorphism. This pattern is characteristic of Grade 3 ccRCC according to WHO/ISUP classification, where prominent nucleoli are a key feature.

The final (“dystrophic”) pattern was characterized by a low number of cells, less than 5000 in a 1 mm^2^ ROI. Patients with this cellular composition had the second worst prognosis, 2.7 years. Tumor growth patterns were alveolar or tubular, with rare hemorrhages, large areas of necrosis, and pronounced edema. Cells with prominent nucleoli were large and sparse, featuring light eosinophilic cytoplasm and large nuclei with irregular borders. Nucleoli were noticeable at ×100 magnification (8.56% ± 7.06%). In some cases where patients died, G3 cells were very rare, indicating that dystrophic processes in tumor tissue interfered with the proliferation of cancer cells (Figure S4). The low cell density indicated extensive dystrophic processes and focal necrosis, particularly the presence of homogeneous clusters of degenerating and dead cells. This pattern correlated with Grades 3 and 4, where nuclear pleomorphism is severe and necrosis is abundant. These findings are in agreement with proposals to incorporate necrosis into the WHO/ISUP grading system to enhance its prognostic accuracy [5, 18, 19].

## 4 Discussion

Our study introduces a data‐driven approach to renal cancer classification, addressing limitations in current grading systems by analyzing tumor cellular profiles. Our findings demonstrate that the presence of Grade 3 (G3) cells strongly correlates with the overall malignancy of the tumor. Importantly, we identified considerable heterogeneity in the cellular composition of tumors currently classified as Grade 3 under the WHO/ISUP system. This variation highlights the limitations of existing grading criteria and the need for a more refined, data‐driven approach that could enable more personalized treatment strategies for ccRCC patients. Moreover, our results underscore the critical prognostic value of tumor cellular composition in understanding disease progression. By identifying four distinct tissue patterns, we demonstrated the prognostic significance of the relative number of cells with prominent nucleoli and the presence of dystrophic changes, such as focal necrosis and edema. These findings suggest that integrating additional morphometric parameters into the WHO/ISUP grading system could improve its prognostic accuracy, supporting more personalized treatment strategies.

Numerous AI‐based models have been developed to support oncopathologists by automating routine tasks such as grading ccRCC. However, many studies oversimplified ccRCC grading into binary groups (low‐grade vs. high‐grade), which undermines the nuanced histological diversity our findings highlight [11, 12, 13, 20]. Previous research employing the Fuhrman grading system demonstrated distinct nuclear size differences, with low‐grade ccRCC nuclei averaging around 6 μm and high‐grade nuclei approximately 9 μm. These size variations were effectively utilized to train AI models that attained high discriminative accuracy (AUC = 0.97) [13]. Another model leveraged nucleolar prominence detection using image pixel intensity metrics and incorporated genetic data to strengthen predictive accuracy, achieving a correlation coefficient of *R* = 0.59 between histological and genetic scores [12]. Kruk et al. applied an ensemble of SVM and random forest models using nuclear texture, geometry, and color, achieving sensitivity and specificity up to 99.3% depending on the grade [20]. AI models, once trained and validated, can also be used to predict survival outcomes based on clinical variables such as age, sex, and TNM stage. For example, one deep learning model applied to 160 ccRCC cases used 26 clinical features and achieved 84.6% sensitivity and 81.3% specificity in predicting tumor grade. In an extended test set, predicted grade was significantly associated with overall survival after adjusting for age and gender (HR = 2.05; 95% CI: 1.21–3.47) [11].

Our investigation presents several conceptual innovations that distinguish it from prior work in the field of AI‐based renal cancer grading. Unlike earlier models that primarily relied on general nuclear morphology or size measurements, we focused specifically on the proportion of tumor cells with prominent nucleoli. To our knowledge, this is the first study to define a prognostically relevant threshold for Grade 3 cells, achieved through precise cell profiling combined with independent survival analysis. We propose a potential integration into clinical workflows by offering a reproducible, interpretable biomarker—namely, the proportion of cells with prominent nucleoli—that can be visually validated within existing digital pathology platforms. Rather than replacing the WHO/ISUP grading system, our approach aims to complement and refine it, providing additional quantitative support for tumor stratification and prognosis estimation.

Although WHO/ISUP grading is widely used in clinical practice, it remains subject to interobserver variability, partly due to the inherently discrete nature of grading systems compared to the continuous spectrum of biological variability. Our AI‐assisted approach addresses this challenge by providing objective, quantitative support for grade determination, based on real morphometric metrics and a statistically validated threshold for the proportion of cells with prominent nucleoli. While interobserver disagreement may persist, our model offers a standardized, reproducible framework that strengthens grading decisions. Importantly, the model was pilot‐integrated into a histological image viewer and tested within a pathology department, demonstrating its potential for practical implementation in a clinical workflow (Video S1). By visually highlighting critical cellular features directly on whole‐slide images, the system facilitates rapid identification of key prognostic patterns without disrupting standard diagnostic procedures. Compared to PD‐L1 expression profiling and genomic biomarkers—which are important but often restricted to specific tumor subtypes or hereditary cancer forms—our approach provides a broadly applicable, morphology‐based prognostic tool. The assessment of nucleolar prominence is a universal feature of tumor biology and thus offers a more generalizable solution for prognostication across diverse cases of ccRCC.

Our work emphasizes the value of analyzing specific morphological features independently of broader histological landscapes. For instance, studies examining cytoplasmic features in frozen ccRCC sections found that cytoplasmic kurtosis in the hematoxylin channel predicted survival [21]. Similarly, quantitative image features extracted with CellProfiler from H&E‐stained slides showed significant survival differences between low‐ and high‐risk patients [22]. These studies, alongside ours, underscore the potential of morphometric analyses to refine existing grading systems.

Limitations of the study include that our AI model focused exclusively on the cells with prominent nucleoli and did not reflect other important morphological criteria of WHO/ISUP classification such as rhabdoid or sarcomatoid dedifferentiation. Although we annotated the tissue according to WHO/ISUP classification, our dataset contained a scarce number of giant multinucleated (G4) cells. As a result, we focused our investigation on a binary classification approach and concentrated on the prognostic value of a specific cell type, G3 cells with prominent nucleoli. The present study used the calculations to predict expert grades and the survival rate of the patients but did not include clinical confounders (age, tumor stage, metastasis, comorbidities, recurrence free survival) or diagnostic data of different modalities (genetic status, immunohistochemistry or radiological measurements). Due to practical constraints, such as the inability to collect multiple formalin‐fixed, paraffin‐embedded tissue blocks from the same case, we were unable to comprehensively evaluate intratumoral heterogeneity. This limitation may have implications for the interpretation of our results, as variations within tumor samples could influence the observed outcomes. Moreover, the presence of different histological staining protocols among various patient cohorts introduces the possibility of color heterogeneity in WSIs. Color variations across WSIs may potentially impact the accuracy and consistency of our analyses. While efforts were made to standardize image processing techniques, these variations remain a concern and should be considered when interpreting our findings. When applying our algorithm, potential errors may arise when analyzing histological scans from laboratories with suboptimal sample preparation quality. To minimize the impact of variations in staining protocols, color normalization techniques should be applied to the digital slides prior to analysis. Additionally, in the presence of artifacts such as folds, tears, or sectioning defects in the paraffin block, it is recommended to implement preprocessing methods, including AI‐based image enhancement and artifact correction, to improve the overall reliability and accuracy of the model's performance.

The survival differences we identified between the “pluralistic” and “nucleolar” patterns (6 years vs. 2.2 years) highlight the need for refined WHO/ISUP criteria. Specifically, our findings suggest that Grade 3 tumors could be more accurately defined by a minimum proportion of G3 cells observed at high magnification. Our analysis of TCGA samples identified a threshold of 11.4%, aligning with our proposed criterion of one in 10 cells showing prominent nucleoli. Furthermore, focal necrosis and edema associated with low cellular density should be considered Grade 4 features in future guidelines.

## 5 Conclusion

Our study introduced a data‐driven classification approach for renal cancer, addressing the limitations of current systems by calculating detailed cellular profiles of tumor regions. By identifying four distinct tumor tissue patterns, we demonstrated that both the relative number of cells with prominent nucleoli and the presence of dystrophic changes, such as focal necrosis and edema, have independent predictive value. These findings indicate that incorporating additional morphometric parameters into future revisions of the WHO/ISUP classification could significantly enhance its prognostic accuracy, enabling more personalized predictions of patient outcomes. This approach offers a promising path for increasing the biological relevance of renal cancer grading, potentially leading to more tailored and effective treatment strategies.

## Author Contributions

Conceptualization, A.F., T.D., and P.T.; methodology, A.F., V.G., M.B., A.A., and P.T.; software, E.I., V.G., D.Z., D.E., M.B., A.T., and A.B.; validation, E.I., A.T., A.B., Y.O., E.R., N.S., A.L., and T.D.; formal analysis, A.F., V.G., and M.B.; investigation, E.I., A.F., V.G., D.Z., D.E., M.B., A.T., A.B., Y.O., E.R., N.S., M.A., and P.T.; resources, D.E., Y.O., E.R., R.P., A.A., N.S., T.D., and P.T.; data curation, E.I., A.F., D.Z., D.E., and M.B.; writing – original draft preparation, E.I., A.F., D.Z., D.E., and M.B.; writing – review and editing, V.G., A.T., A.B., Y.O., E.R., A.A., R.P., M.A., A.L., T.D., and P.T.; visualization, E.I., A.F., V.G., D.Z., D.E., and M.B.; supervision, P.T.; project administration, A.F., Y.O., E.R., A.A., A.L., T.D., and P.T.; funding acquisition, P.T.

## Ethics Statement

All experimental protocols were approved by Sechenov University (Moscow, Russia).

## Consent

Informed consent was obtained from all subjects and/or their legal guardian(s) prior to having their data used in this study.

## Conflicts of Interest

Victor Grinin, Dmitry Zhavoronkov, Dmitry Ermilov and Alexander Arutyunyan have received funding for theirs work through employment at PJSC VimpelCom. Ruslan Parchiev has received funding for his work through employment at Medical Neuronets. All the other authors declared no competing interests.

## Supporting information


**Figure S1:** Validation Confusion Matrix of Grade 3 Cell Classification. This confusion matrix illustrates the performance of our classification model in distinguishing between Grade 3 cells (True = 1) and other cells (True = 0).
**Figure S2:** Images of cells that were misclassified as Grade 3 by the trained model. Cells were identified as Grade 3 by the model; however, they had an expert label different from G3. Most of these cells were blurry, however, they had one or several nucleoli and were labeled as G3^−^ due to their imperfect image or stellate shape of the nucleoli.
**Figure S3:** Confusion matrix for misclassified samples in the validation set. The model demonstrates high precision in identifying cells with prominent nucleoli (G3+), with minimal misclassification of lower‐grade cells (G1+, G2+).
**Figure S4:** Identification of cells with prominent nucleoli (G3 cells, highlighted yellow) in Grade 1 and Grade 2 cases. The relative content of G3 cells in Grade 1 and Grade 2 ccRCC cases was lower than in Grades 3 and 4; however, they were still present. These cells were typically large, round and exhibited nucleoli visible at low magnification.
**Figure S5:** Illustration of sparse distribution of cells (including cells with prominent nucleoli, highlighted yellow) in dystrophic tissue pattern. Cells with prominent nucleoli were locater far from each other due to edema and focal necrosis. Dystrophic processes in tumor tissue interfered with proliferation of cancer cells.

**Video S1:** Demonstration of the AI model integrated into a digital histology viewer.

## Data Availability

The datasets generated during and/or analyzed during the current study are available from the corresponding author on reasonable request.
