# Supplementary material for: Predicting Grade and Patient Survival in Renal Cancer Using Machine Learning Analysis of Nucleolar Prominence
Source: Cancer Med. 2025 Sep 9;14(17):e71196. doi: 10.1002/cam4.71196 (PMC12420676; doi:10.1002/cam4.71196)
Supplement: Supplementary file 1 — Figure S1: Validation Confusion Matrix of Grade 3 Cell Classification. This confusion matrix illustrates the performance of our classification model in distinguishing between Grade 3 cells (True = 1) and other cells (True = 0). Figure S2: Images of cells that were misclassified as Grade 3 by the trained model. Cells were identified as Grade 3 by the model; however, they had an expert label different from G3. Most of these cells were blurry, however, they had one or several nucleoli and were labeled as G3− due to their imperfect image or stellate shape of the nucleoli. Figure S3: Confusion matrix for misclassified samples in the validation set. The model demonstrates high precision in identifying cells with prominent nucleoli (G3+), with minimal misclassification of lower‐grade cells (G1+, G2+). Figure S4: Identification of cells with prominent nucleoli (G3 cells, highlighted yellow) in Grade 1 and Grade 2 cases. The relative content of G3 cells in Grade 1 and Grade 2 ccRCC cases was lower than in Grades 3 and 4; however, they were still present. These cells were typically large, round and exhibited nucleoli visible at low magnification. Figure S5: Illustration of sparse distribution of cells (including cells with prominent nucleoli, highlighted yellow) in dystrophic tissue pattern. Cells with prominent nucleoli were locater far from each other due to edema and focal necrosis. Dystrophic processes in tumor tissue interfered with proliferation of cancer cells. [file CAM4-14-e71196-s001.docx]

**Fig. S1. Validation Confusion Matrix of Grade 3 Cell Classification.** This confusion matrix illustrates the performance of our classification model in distinguishing between Grade 3 cells (True=1) and other cells (True=0)

**
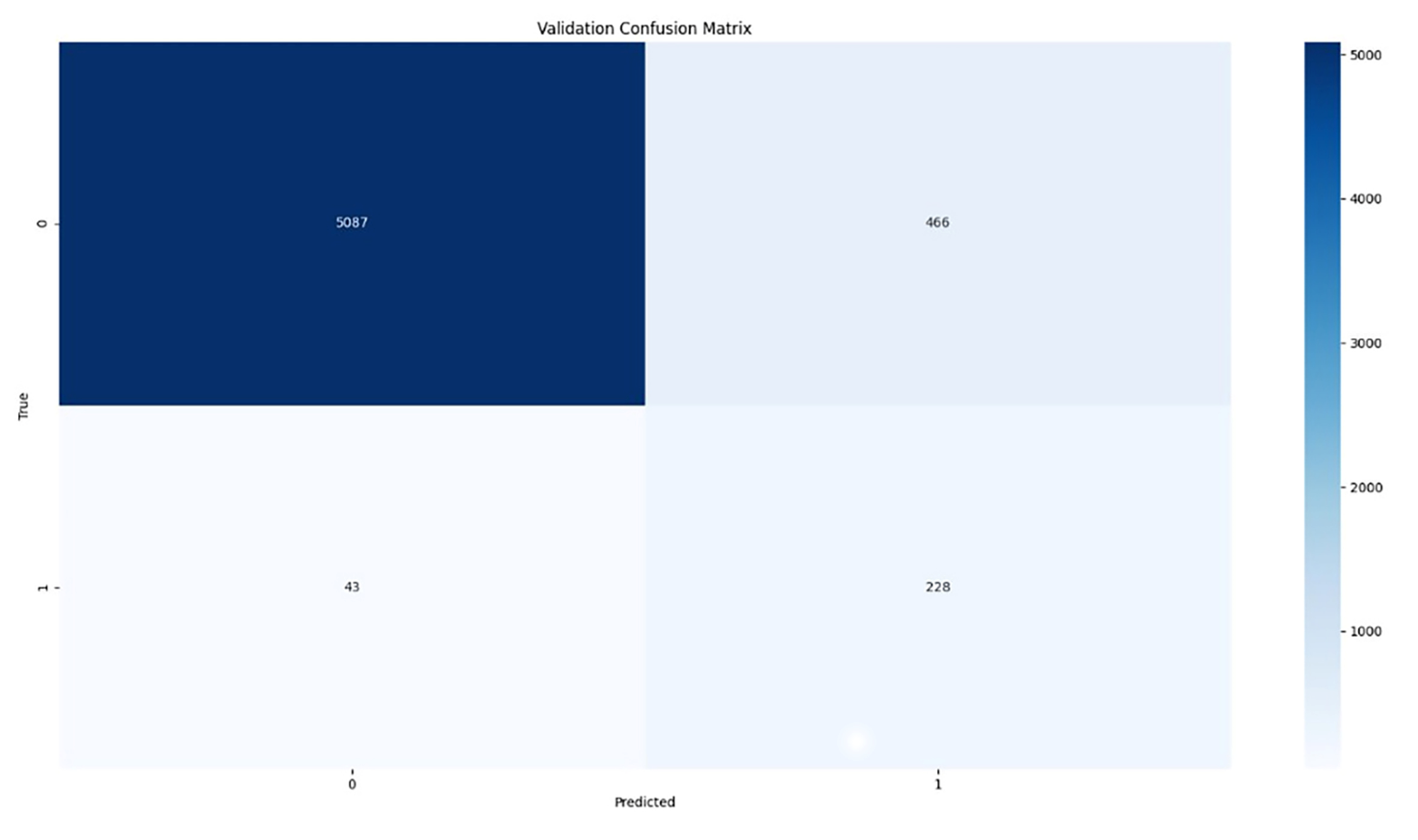
**

**Fig. S2. Images of cells that were misclassified as Grade 3 by the trained model.** Cells were identified as Grade 3 by the model; however, they had an expert label different from G3. Most of these cells were blurry, however, they had one or several nucleoli and were labeled as G3^-^ due to their imperfect image or stellate shape of the nucleoli


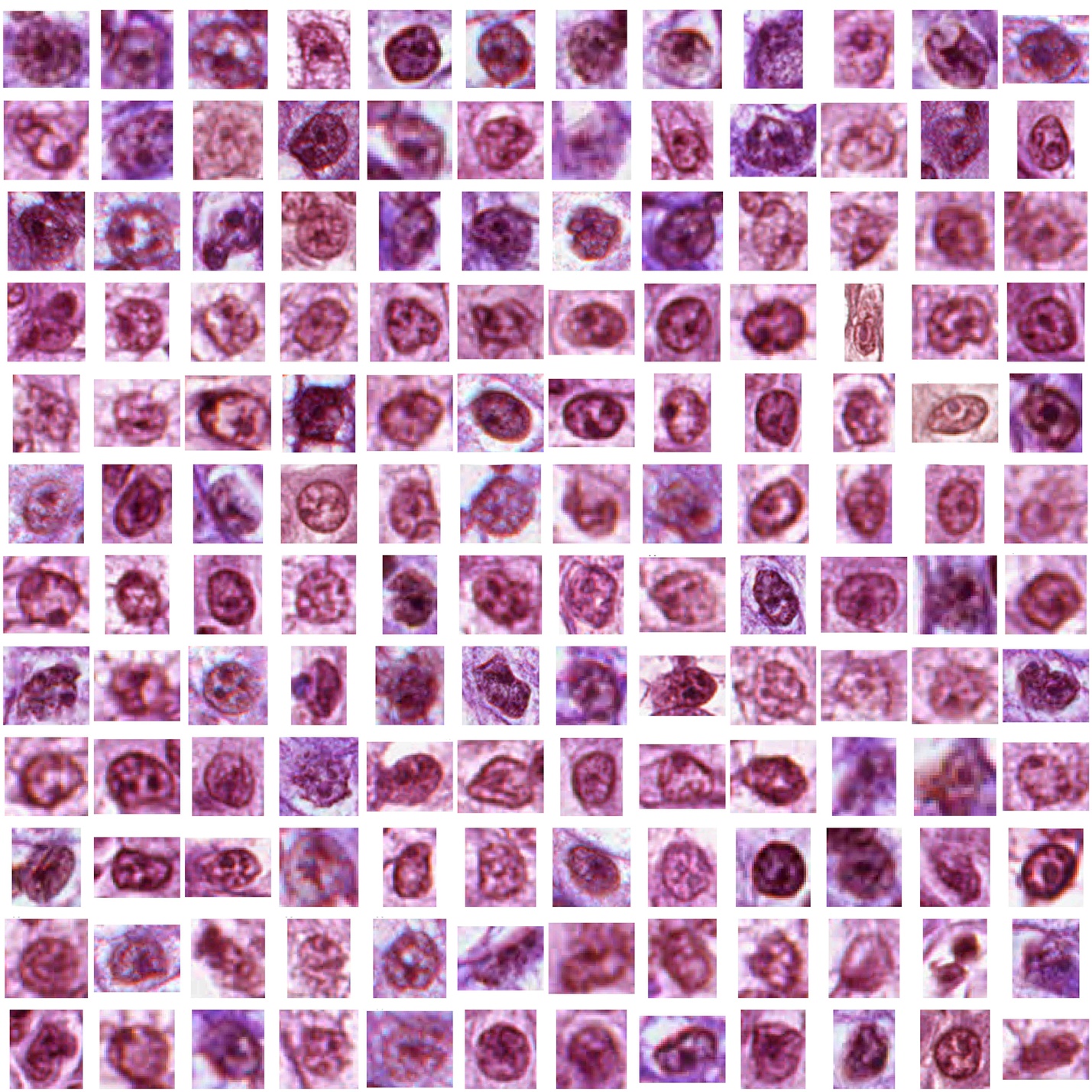


**Fig. S3. Confusion matrix for misclassified samples in the validation set.** The model demonstrates high precision in identifying cells with prominent nucleoli (G3+), with minimal misclassification of lower-grade cells (G1+, G2+).


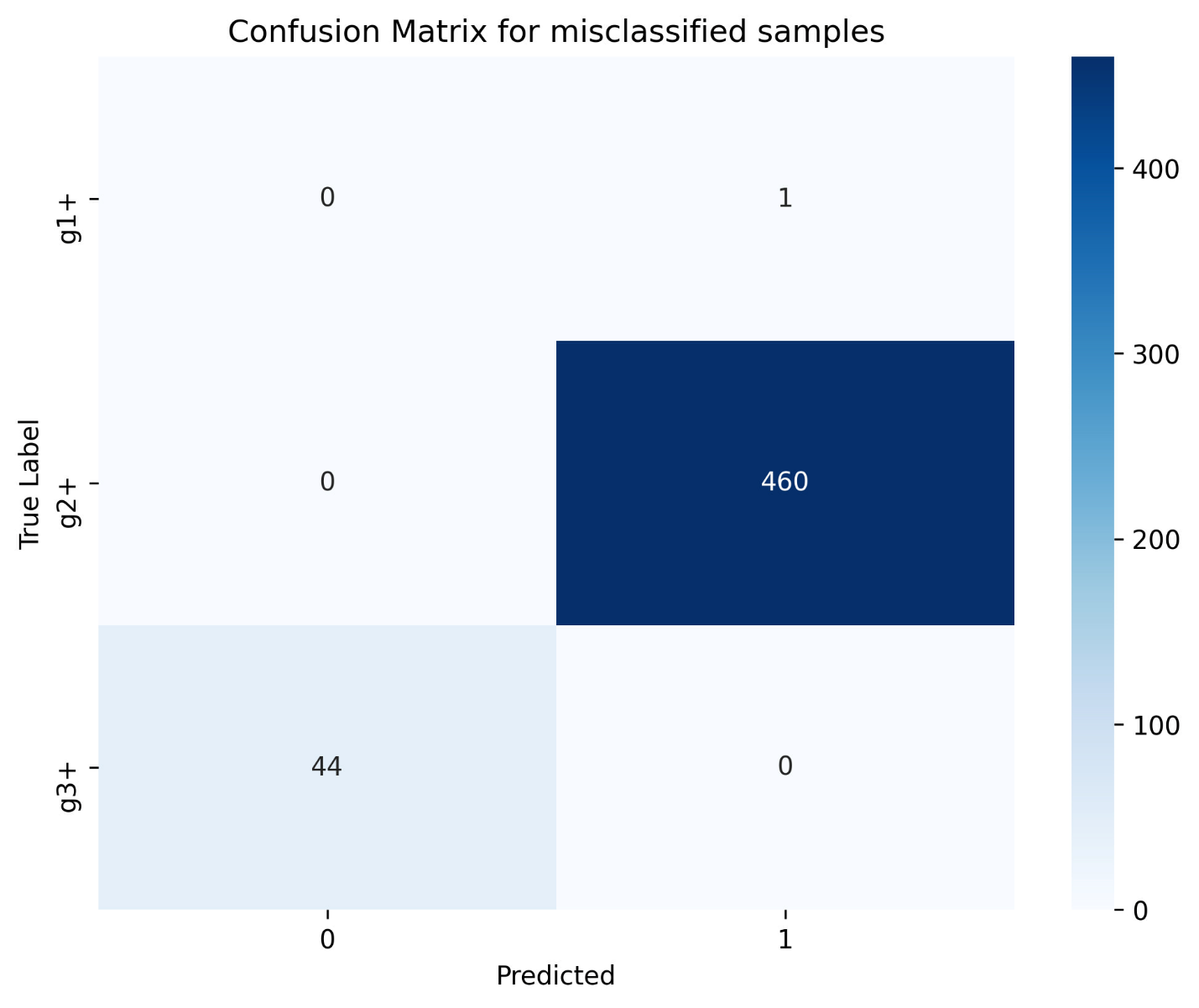


**Fig. S4. Identification of cells with prominent nucleoli (G3 cells, highlighted yellow) in Grade 1 and Grade 2 cases.** The relative content of G3 cells in Grade 1 and Grade 2 ccRCC cases was lower than in Grades 3 and 4; however, they were still present. These cells were typically large, round and exhibited nucleoli visible at low magnification


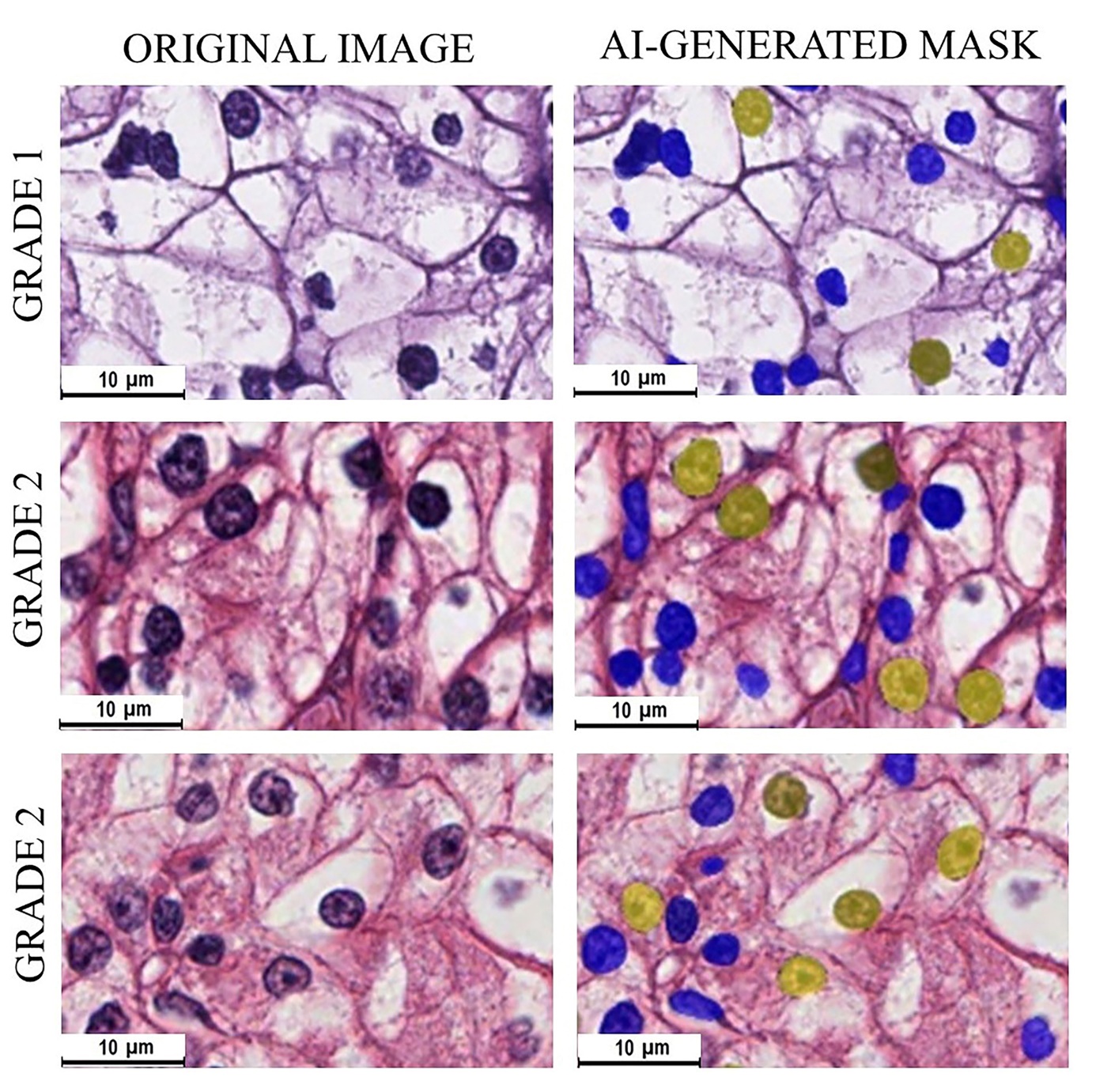


**Fig. S5. Illustration of sparse distribution of cells (including cells with prominent nucleoli, highlighted yellow) in dystrophic tissue pattern.** Cells with prominent nucleoli were locater far from each other due to edema and focal necrosis. Dystrophic processes in tumor tissue interfered with proliferation of cancer cells





**Video S1. Demonstration of the AI model integrated into a digital histology viewer.**

**
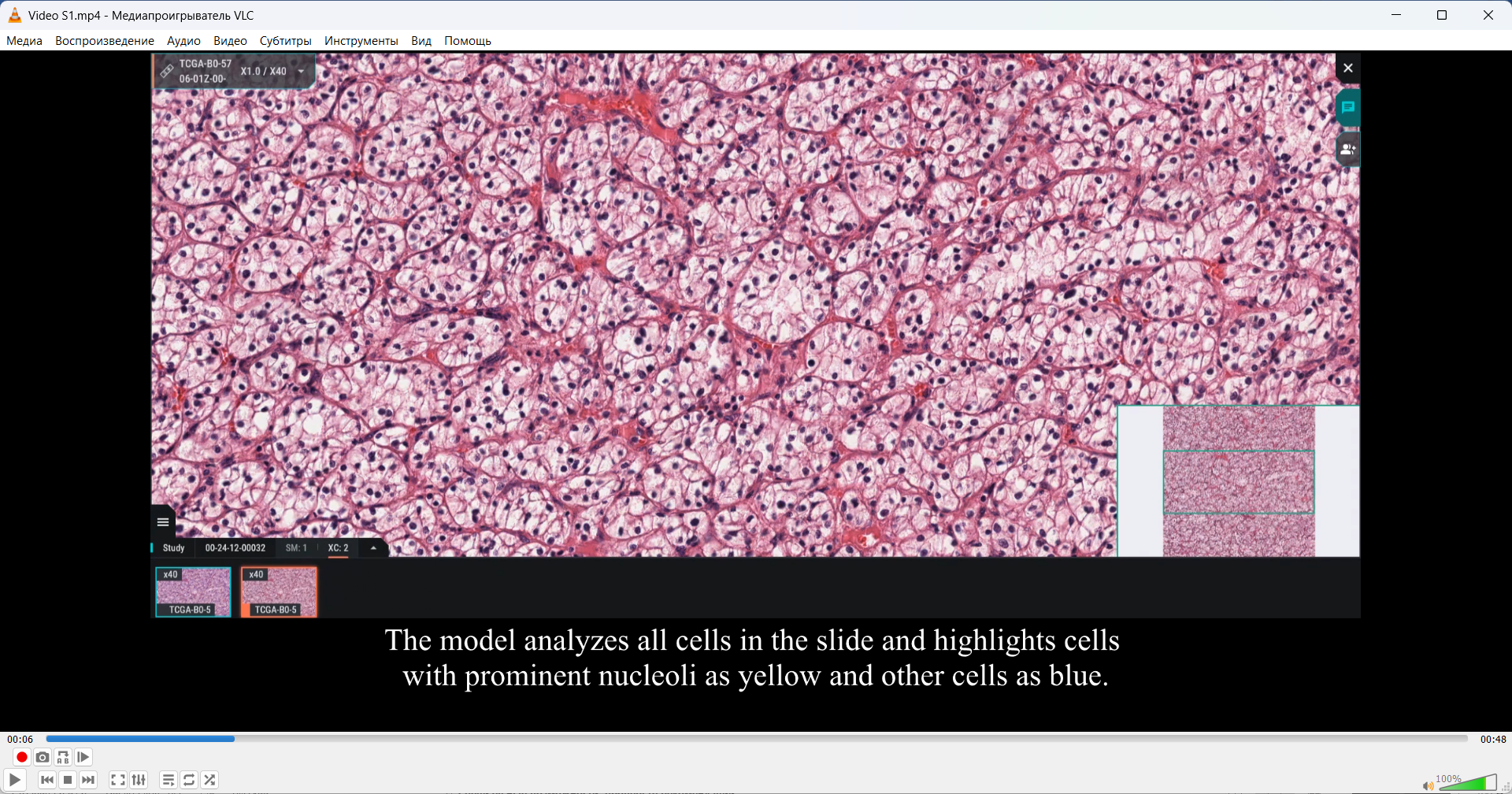
**
